# Handling informative dropout in longitudinal analysis of health-related quality of life: application of three approaches to data from the esophageal cancer clinical trial PRODIGE 5/ACCORD 17

**DOI:** 10.1186/s12874-020-01104-w

**Published:** 2020-09-03

**Authors:** B. Cuer, C. Mollevi, A. Anota, E. Charton, B. Juzyna, T. Conroy, C. Touraine

**Affiliations:** 1 Biometrics Unit, Montpellier Cancer Institute (ICM), University of Montpellier, 208, avenue des Apothicaires, 34298 Montpellier, France; 2 French National Platform Quality of Life and Cancer, Montpellier, France; 3 Institute of Cancer Research of Montpellier (IRCM)- Inserm 1194, ICM, University of Montpellier, Montpellier, France; 4 Methodology and Quality of Life Unit in Oncology- Inserm UMR 1098, University Hospital of Besançon, Besançon, France; 5 University Bourgogne Franche-Comté, Inserm, EFS BFC, UMR 1098, Interactions Hôte-Greffon-Tumeur/Ingénierie Cellulaire et Génique, Besançon, France; 6 UNICANCER R&D, French Federation of Comprehensive Cancer Centres, Paris, France; 7 Department of Medical Oncology, Institut de Cancérologie de Lorraine, Vandoeuvre-lès-Nancy, France; 8 APEMAC, équipe MICS, Université de Lorraine, Nancy, France

**Keywords:** Pattern-mixture model, Selection model, Shared-parameters model, Joint modeling, Health-related quality of life, Informative dropout, Cancer clinical trial

## Abstract

**Background:**

Health-related quality of life (HRQoL) has become a major endpoint to assess the clinical benefit of new therapeutic strategies in oncology clinical trials. Typically, HRQoL outcomes are analyzed using linear mixed models (LMMs). However, longitudinal analysis of HRQoL in the presence of missing data remains complex and unstandardized. Our objective was to compare the modeling alternatives that account for informative dropout.

**Methods:**

We investigated three alternative methods—the selection model (SM), pattern-mixture model (PMM), and shared-parameters model (SPM)—in relation to the LMM. We first compared them on the basis of methodological arguments highlighting their advantages and drawbacks. Then, we applied them to data from a randomized clinical trial that included 267 patients with advanced esophageal cancer for the analysis of four HRQoL dimensions evaluated using the European Organisation for Research and Treatment of Cancer (EORTC) QLQ-C30 questionnaire.

**Results:**

We highlighted differences in terms of outputs, interpretation, and underlying modeling assumptions; this methodological comparison could guide the choice of method according to the context. In the application, none of the four models detected a significant difference between the two treatment arms. The estimated effect of time on HRQoL varied according to the method: for all analyzed dimensions, the PMM estimated an effect that contrasted with those estimated by the SM and SPM; the LMM estimated effects were confirmed by the SM (on two of four HRQoL dimensions) and SPM (on three of four HRQoL dimensions).

**Conclusions:**

The PMM, SM, or SPM should be used to confirm or invalidate the results of LMM analysis when informative dropout is suspected. Of these three alternative methods, the SPM appears to be the most interesting from both theoretical and practical viewpoints.

**Trial registration:**

This study is registered with ClinicalTrials.gov, number NCT00861094.

## Background

Health-related quality of life (HRQoL) is often a secondary endpoint in cancer clinical trials. It is also increasingly being used as a primary or co-primary endpoint [1]. HRQoL is assessed at different time points throughout the care process (at baseline, during treatment, and during follow-up) by self-administered questionnaires composed of items assessing different HRQoL dimensions. The HRQoL outcome to be analyzed consists of longitudinal dimension-specific score data. However, the rate of completed questionnaires generally decreases over time and, in addition, some items may be missing among available questionnaires. This leads to missing data that are said to be monotone if the score is not available from a certain time point until the end of the study, and intermittent otherwise. The nature of the missing data mechanism depends on how the missingness is related to the HRQoL outcome.

Missing data are classified as missing completely at random (MCAR) if missingness is independent of the (observed or unobserved) HRQoL outcome or depends only on observed characteristics, as missing at random (MAR) if missingness additionally depends on the observed HRQoL outcome, and as missing not at random (MNAR) if missingness is dependent of the unobserved HRQoL outcome [2, 3]. The terms informative or non-ignorable are also used to refer to MNAR data. In the presence of incomplete longitudinal outcome data, the strategy of analysis should be adapted to the nature of the missing data mechanism in order to avoid biased or inaccurate results. In most studies, the missing data mechanism is not characterized, so methods used to analyze longitudinal HRQoL data in randomized clinical trials [4] are potentially inadequate.

Linear mixed models (LMMs) are powerful and flexible models for the analysis of repeated measures of a continuous outcome. This class of model is classically used to compare changes in HRQoL over time between experimental and control arms in cancer clinical trials [5, 6]. However, the occurrence of intermittent or monotone missing data could compromise the longitudinal analysis of HRQoL data, leading to a loss of statistical power at best, and, at worse, biased estimates; for instance, in palliative or advanced disease situations, where missing data could be related to the health status of patients too ill to complete their HRQoL questionnaires [7, 8]. Likelihood-based methods that use all the observed information (as in LMMs) are valid when the missing data are MAR [9]. However, in the presence of informative missing data (i.e., MNAR), the two processes that are the longitudinal HRQoL outcome and the missing data mechanism have to be jointly modeled to prevent a biased estimation [10, 11].

Since the end of the 1980s, different models have been proposed for the joint distribution of the longitudinal outcome and the missingness process. More attention has been devoted to monotone missing data, corresponding to dropout, which is more likely to be informative and generally easier to handle. Pattern-mixture models (PMMs) and selection models (SMs) are based on the two possible decompositions of the joint distribution [12, 13]. In recent years, the joint models or shared-parameter models (SPMs), where the association between the two processes is captured by shared parameters, have received much attention [14, 15]. In clinical trials, SPMs are mostly used to jointly analyze a longitudinal outcome and overall survival. They can also be used to take into account and study the relationship between a longitudinal HRQoL outcome and time-to-dropout [16].

There are relatively few publications that compare these three approaches from a perspective of their practical application to clinical trial data [17–19]. This is needed to further our understanding of their use and interpretation; the insufficient knowledge about these models could explain why they are rarely used in clinical trials.

The objectives of this paper were to compare the PMM, the SM, and the SPM with each other and then to compare these models with the LMM, for the analysis of an HRQoL outcome in the presence of informative dropout. First, we compare the models from a methodological point of view, highlighting the advantages and drawbacks of each one. Then, we illustrate and interrogate them in the longitudinal analysis of four HRQoL dimensions in patients with advanced esophageal cancer from the PRODIGE 5/ACCORD 17 clinical trial.

## Methods

We highlighted the differences between the PMM, SM, and SPM in handling informative dropout when analyzing a longitudinal HRQoL outcome and interpreted their results in relation to those from the LMM. For this purpose, we first made a methodological comparison of the four models by highlighted their differences in terms of underlying modelling assumptions and interpretation. The advantages and drawbacks of each of model are then illustrated through an analysis of data from the PRODIGE 5/ACCORD 17 clinical trial (NCT00861094).

### Illustrative clinical trial

#### Study design

In the PRODIGE 5/ACCORD 17 clinical trial, 267 patients with advanced esophageal cancer were randomly assigned to either an experimental arm (*N* = 134) receiving a FOLFOX (fluorouracil plus leucovorin and oxaliplatin) regimen or a control arm (*N* = 133) receiving a fluorouracil and cisplatin regimen as part of chemoradiotherapy treatment. The primary endpoint was progression-free survival and one of the secondary endpoints was HRQoL. The statistical analysis of the primary endpoint revealed no significant difference between the two treatment arms. More details concerning inclusion and exclusion criteria, study design, protocol treatment, HRQoL assessment, and compliance have been previously published [20, 21].

#### HRQoL assessment

HRQoL was prospectively assessed using the European Organisation for Research and Treatment of Cancer (EORTC) Quality of Life Questionnaire Core 30 (QLQ-C30, version 3.0) [22] at baseline, during treatment (months 1.25 and 3), at month 4, and after treatment during follow-up (at months 6, 12, 24, and 36). This self-administered questionnaire contains 30 items evaluating five functional scales, nine symptomatic scales/items, and one global health status/HRQoL scale. Standardized scores from 0 to 100 can be calculated for each scale according to the scoring procedure recommended by the EORTC [23]. A high score for the functional and global health status scales corresponds to good functional capacities and reflects a high level of HRQoL, whereas a high score for the symptom scales corresponds to a high level of symptoms and reflects a poor HRQoL. Four dimensions were pre-specified in the protocol as targeted dimensions: global health status/HRQoL (QL scale), physical functioning (PF scale), pain (PA scale), and fatigue (FA scale). In what follows, we will consider only these four dimensions (or scales).

### Statistical analysis

All analyses were performed in the evaluable intent-to-treat population: a patient was considered as evaluable for a given scale when the score was available at least once during the study, whatever the corresponding measurement time. We used the four models described below in Eqs. (1), (3), (5) and (8) to analyze the longitudinal HRQoL score data conditionally to baseline covariates in the presence of potentially informative monotone missing data (dropout).

We first used the LMM that is valid under the MAR assumption. We then modeled the joint distribution of the longitudinal outcome and the dropout process using three models that are valid under the MNAR assumption: the SM and the PMM, which are based on the two existing and converse factorizations of the joint distribution, and the SPM, where the longitudinal outcome and the time-to-dropout are linked through a function of the random effects. In these three models, we used the LMM presented below as the sub-model for the HRQoL score.

#### Linear mixed model (LMM)

We modeled the HRQoL score trajectories by a random coefficients LMM. The HRQoL score for patient *i* at time *t*_*j*_ of the *j-*th planned visit was expressed as follows:

1 $$ {Y}_i\left({t}_j\right)={\beta}_0+{\beta}_1{t}_j+{\beta}_2\left\{ ar{m}_i\times {t}_j\right\}+{b}_{0i}+{b}_{1i}{t}_j+{\varepsilon}_i\left({t}_j\right) $$

where *arm*_*i*_ is the arm indicator variable for patient *i* (0: control, 1: experimental), *β*_0_ is the intercept, *β*_1_ the slope in the control arm, and *β*_2_ the interaction effect corresponding to the difference between the slopes in the experimental and control arms. With this parametrization, the quantity *β*_1_ + *β*_2_ represents the slope in the experimental arm. The random intercept *b*_0*i*_ and the random slope *b*_1*i*_ take into account the repeated measurements on the same patient and correspond to the individual deviations from the fixed intercept and slope, respectively. They are assumed to be normally distributed with a mean of 0 and a 2 × 2 unconstrained covariance matrix to estimate. The error term denoted by *ε*_*i*_(*t*_*j*_) is also assumed to be normally distributed with a mean of 0 and a variance to estimate.

In what follows, *Y*_*i*_, *X*_*i*_, and *D*_*i*_ denote respectively the vector of longitudinal HRQoL scores, the vector of covariates, and the dropout variable for patient *i*.

#### Selection model (SM)

The SM is based on the decomposition of the joint distribution into the marginal distribution of the HRQoL score and the conditional distribution of the dropout variable given the HRQoL score:

2 $$ f\left({Y}_i,{D}_i\mid {X}_i\right)=f\left({Y}_i\mid {X}_i\right)\times f\left({D}_i\mid {Y}_i,{X}_i\right) $$

where the dropout variable *D*_*i*_ corresponds to the visit at which the last available HRQoL assessment took place, i.e., before patient *i* dropout. In cases of no dropout, *D*_*i*_ = *J*, where *J* is the number of planned visits. We modeled the HRQoL score using the LMM in Eq. (1). We modeled the conditional probability of dropout at each visit *j* = 1, …, *J* by the logistic regression proposed by Diggle and Kenward [24]:

3 $$ logit\left[P\left({D}_i=j\mid {D}_i\ge j,{Y}_i\left({t}_j\right),{Y}_i\left({t}_{j+1}\right)\right)\right]={\psi}_0+{\psi}_1{Y}_i\left({t}_j\right)+{\psi}_2{Y}_i\left({t}_{j+1}\right) $$

The dropout probability is allowed to depend on the last (observed) HRQoL score *Y*_*i*_(*t*_*j*_) and the current (unobserved) HRQoL score *Y*_*i*_(*t*_*j* + 1_). A non-zero parameter *ψ*_1_ would be in favor of the MAR assumption and a non-zero parameter *ψ*_2_ in favor of the MNAR assumption (informative dropout). If only the *ψ*_0_ parameter is non-zero, the dropout can be considered to be independent of the HRQoL score (MCAR assumption).

#### Pattern-mixture model (PMM)

The PMM is based on the other possible decomposition of the joint distribution, that is, the decomposition into the marginal distribution of the dropout variable and the conditional distribution of the HRQoL score given the dropout variable:

4 $$ f\left({Y}_i,{D}_i\mid {X}_i\right)=f\left({D}_i\mid {X}_i\right)\times f\left({Y}_i\mid {D}_i,{X}_i\right) $$

where the dropout variable corresponds to the pattern of missing data: *D*_*i*_ = *k*, *k* = 1, …, *K*, where *K* is the number of possible patterns. In the simplest case, the variable is defined as a dropout indicator (*K* = 2); in the most complex case, the variable is defined as the number of dropout possibilities: *D*_*i*_ = *k*, *k* = 1, …, *J*, where *J* is the number of planned visits. In our application, we classified a patient as belonging to a certain pattern when she/he dropped out within a specific time interval covering one or several visits.

In the PMM, a multinomial distribution is assumed for the dropout probability, meaning that the probability of belonging to pattern *k* is simply estimated by the proportion *π*_*k*_ of patients belonging to pattern *k*.

We modeled the conditional HRQoL score trajectory using an LMM similar to the LMM in Eq. (1) in each pattern *k*:

5 $$ {Y}_{ik}\left({t}_j\right)={\beta}_0^k+{\beta}_1^k{t}_j+{\beta}_2^k\ \left\{ ar{m}_{ik}\times {t}_j\right\}+{b}_{0 ik}+{b}_{1 ik}{t}_j+{\varepsilon}_{ik}\left({t}_j\right) $$

Note that in the PMM approach, the fixed effects differ according to the dropout pattern. The following formula allows estimates to be obtained for the marginal distribution of the HRQoL score (irrespective of the pattern):

6 $$ {\beta}_l=\sum \limits_{k=1}^K{\beta}_l^k{\pi}_k,\kern0.5em l=0,1,2 $$

It corresponds to a weighted sum of the pattern-specific parameters. Confidence intervals can then be calculated using the delta method [25].

#### Shared-parameter model (SPM)

The SPM captures the association between the time-to-dropout and the longitudinal HRQoL outcome through shared parameters that include the random effects *b*_*i*_, so that the HRQoL score and the dropout variable are supposed to be conditionally independent given the random effects:

7 $$ {\displaystyle \begin{array}{l}f\left({Y}_i,{D}_i|{X}_i\right)=\int f\left({Y}_i,{D}_i|{X}_i\right){\mathrm{d}b}_i\\ {}=\int f\left({Y}_i|{b}_i,{X}_i\right)\times f\left({D}_i|{b}_i,{X}_i\right)\times f\left({b}_i\right){\mathrm{d}b}_i\end{array}} $$

where the dropout variable *D*_*i*_ corresponds to a time-to-dropout variable. In our application, dropout is not related to an event occurring at any time but corresponds to non-response after a certain visit. Thus, we defined *D*_*i*_ as the delay between inclusion and the last visit in which HRQoL assessment occurred. We modeled the HRQoL score using the LMM in Eq. (1). We modeled the risk of dropout at time *t*_*j*_ using a Cox-type survival model.

In the SPM, the association between the HRQoL score and dropout is modeled by including a function of the variables and parameters from the model for *Y*_*i*_ as a time-dependent variable in the survival model. We used the current value parametrization, which means that the time-dependent variable corresponded to the true current HRQoL score value: ${Y}_i^{\star}\left({t}_j\right)={Y}_i\left({t}_j\right)-{\varepsilon}_i\left({t}_j\right).$ More precisely, we used the following model for *D*_*i*_:

8 $$ {\lambda}_i\left({t}_j\mid ar{m}_i\right)={\lambda}_0(t)\mathit{\exp}\left\{\gamma ar{m}_i+\alpha {Y}_i^{\star}\left({t}_j\right)\right\} $$

where *λ*_0_ is the baseline hazard function, *γ* denotes the arm effect on the instantaneous risk of dropout, and *α* is the parameter that quantifies the association between risk of dropout and true current HRQoL score.

#### Statistical software

We fitted the four models to the PRODIGE 5/ACCORD 17 data using the R software (code available on request). For LMM estimation, we used the restricted maximum likelihood method (REML) from the R package nlme [26]. The SM was not available in standard statistical software and required sophisticated programming: the Diggle and Kenward model involved marginalization over the unobserved outcomes and the computation of the likelihood required evaluation of integrals approximated by the Romberg numerical algorithm. We implemented a maximum likelihood function procedure based on a Newton-type algorithm. To apply the PMM required that we apply an LMM with indicator variables for the pattern. We then combined the PMM estimates following Eq. (6) to obtain marginal estimates and implemented a delta method to obtain their confidence intervals. For the SPM, we used the R package JM [27] by assuming a piecewise-constant function for the baseline hazard λ_0_ with seven intervals for the baseline (six internal knots placed at months 1.25, 3, 4, 6, 12, and 24) and the pseudo-adaptive Gauss-Hermite method with nine quadrature points to approximate the integrals over the random effects.

## Results

### Methodological comparison

Table 1 compares the four approaches (LMM, SM, PMM, and SPM) from a methodological point of view.

**Table 1:** Methodological comparison of the four models used for analysis of longitudinal HRQoL score data

	LMM	SM	PMM	SPM
**MODELING**				
**Validity of the model**	Under MAR assumption	Under MNAR assumption	Under MNAR assumption	Under MNAR assumption
**Model for the HRQoL outcome** ***Y***	LMM	LMM	LMM by pattern	LMM
**Model for the dropout variable**	–	Logistic Dropout at specific time (discrete)	Multinomial Dropout at specific time (discrete)	Survival model Dropout at any time (continuous)
**Graphical outputs**	Mean HRQoL score over time according to treatment arm	Mean HRQoL score over time according to treatment arm	(Mean HRQoL score over time according to treatment arm) Mean HRQoL score over time according to treatment arm for each dropout pattern	Mean HRQoL score over time according to treatment arm Hazard function of dropout according to treatment arm
**ESTIMATIONS AND INTERPRETATION**				
**Main estimated parameters**	Fixed effects (*β*_0_, *β*_1_, and *β*_2_)	Fixed effects (*β*_0_, *β*_1_, and *β*_2_) Logistic regression coefficients (*ψ*_0_, *ψ*_1_, and *ψ*_2_)	(Fixed effects overall patterns (*β*_0_, *β*_1_, and *β*_2_)) Fixed effects in each pattern *k* (${\beta}_0^k$, ${\beta}_1^k$, and ${\beta}_2^k$) Proportion in each pattern (*π*_*k*_)	Fixed effects (*β*_0_, *β*_1_, and *β*_2_) Association parameter (*α*) Effect of arm on instantaneous risk of dropout (*γ*)
**Interpretation**	Improvement/deterioration of the HRQoL	Improvement/deterioration of the HRQoL Testing MNAR assumption: a non-null *ψ*_2_ when probability of dropout is associated with unobserved *Y*	(Improvement/deterioration of the HRQoL) Improvement/deterioration of the HRQoL in each dropout pattern	Improvement/deterioration of the HRQoL Risk of dropout over time Testing MNAR assumption: a non-null *α* when instantaneous risk of dropout is associated with current value of *Y*
**Underlying assumptions**	–	Normality of the complete (observed *and* unobserved) *Y*	Extrapolation of the conditional distribution of *Y* (given the dropout pattern) beyond the dropout to obtain estimations for the marginal distribution of *Y*	Conditional independence of *Y* and *T* given the random effects Normality assumption of the random effects distribution
**Key limitations**	Do not account for informative dropout	Dropout in discrete time Not directly available in classical statistical software	Dropout in discrete time Do not directly provide marginal estimates	Computationally challenging to approximate integrals over random effects
**Main software**	R (nlme) SAS (PROC MIXED) Stata (mixed)	S plus (OSWALD, pcmid function but not currently available) Implemented with R in our application (sophisticated programming)	Implemented with R in our application (easy programming)	R (JM, JMBayes) SAS (%JM) Stata (stjm)

Legend: *LMM* Linear Mixed Model, *SM* Selection Model, *PMM* Pattern-Mixture Model, *SPM* Shared-Parameter Model, *MCAR* Missing Completely At Random; *MAR* Missing At Random, *MNAR* Missing Not At Random, *HRQoL* Health-Related Quality of Life

In cases of non-informative dropout (MAR assumption), the likelihood-based LMM that uses all observed data provides valid results; in cases of informative dropout (MNAR assumption), the risk of dropout needs to be modeled using one of the three other approaches.

The SM explains the probability of dropout by a logistic regression; the PMM estimates the probability of belonging to a certain pattern of dropout with a multinomial distribution; the SPM uses a survival model for the time-to-dropout. The SM and PMM suppose that dropout occurs at the discrete assessment times of the HRQoL. By contrast, the SPM treats the time variable as continuous, making it possible to take into account the fact that the dropout could arise at any time during the study.

The fixed parameters *β*_0_, *β*_1_, and *β*_2_ characterizing the mean HRQoL score trajectories are directly estimated using the LMM, SM, and SPM, or obtained indirectly by extrapolation using the PMM. More precisely, the PMM estimates the HRQoL score trajectory parameters at the level of each pattern *k*; afterwards, marginal estimates can be calculated as weighted averages using the proportion *π*_*k*_ of patients in each dropout pattern. Note that this calculation implicitly extrapolates the HRQoL score trajectories beyond the dropout. Thus, all models can be used to graphically represent the mean HRQoL score over time according to treatment arm, directly (LMM, SM, SPM) or indirectly (PMM). The PMM provides complementary graphs specific to the dropout pattern, which can be useful to understand and visualize how the risk of dropout is linked to the HRQoL. The SPM allows a graphical representation of the risk of dropout over time. The informative nature of the dropout can also be tested using additional parameters of the SM or SPM: the *ψ*_2_ coefficient in the logistic regression of the SM indicates how the probability of the HRQoL score to be missing at a certain time depends on the missing value at this time, while the *α* coefficient in the Cox regression of the SPM indicates how the instantaneous risk of dropout at any time is associated with the current HRQoL score.

Nevertheless, the models used to study the evolution of HRQoL scores in the presence of informative dropout require additional assumptions that are untestable on the basis of the observed data. We have already mentioned extrapolating the HRQoL trajectories beyond the dropout in the PMM. The SM is based on the assumption of a normal distribution of the complete (i.e., observed and unobserved) HRQoL score variable. The SPM assumes independence between the longitudinal outcome and dropout process conditionally to the random effects.

The estimates of each model can be obtained using usual statistical software (including R, SAS, and Stata). Specific software has already been developed for LMM and SPM. However, applying the SM and the PMM requires a programming effort. In particular, applying the SM requires implementation and maximization of the likelihood function.

### Application on data from the PRODIGE 5/ACCORD 17 clinical trial

#### Monotone missing data in HRQoL outcomes

**Fig. 1:**
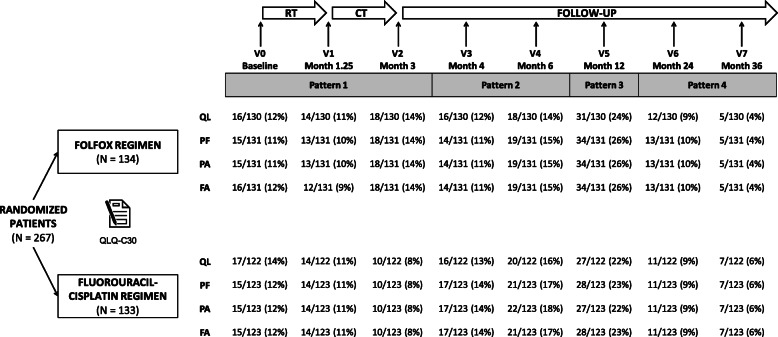
Patients who dropped out after visits V0 to V6, or did not drop out (V7). Legend: Ratio calculated by treatment arm in evaluable patients for each of the four dimensions of EORTC QLQ-C30 (QL, global health status; PF, physical functioning; PA, pain; and FA, fatigue) during radiochemotherapy (RT), chemotherapy (CT), and follow-up visits (V)

At each scheduled visit, there were missing HRQoL score data. From the 267 patients of the intent-to-treat population (experimental arm: *N* = 134; control arm: *N* = 133), the remaining evaluable patients, i.e., with at least one available HRQoL score, were 252 for scale QL (experimental arm: *N* = 130; control arm: *N* = 122), and 254 for scales PF, PA and FA (experimental arm: *N* = 131; control arm: *N* = 123). In fact, the proportion of available scores for scales QL, PF, PA, and FA decreased over time, mostly because of monotone missing data that can be attributed to dropouts (see Fig. 1). For example, for the QL scale, 16/130 patients (12%) in the experimental arm and 17/122 patients (14%) in the standard arm dropped out after the baseline visit (V0, baseline); at the last scheduled visit (V7, month 36), 125/130 patients (96%) in the experimental arm and 115/122 patients (94%) in the standard arm had dropped out (i.e., only 5/130 (4%) and 7/122 (6%) patients completed the questionnaire or the items associated with the QL scale until V7). The distribution of the dropouts seemed homogeneous in both treatment arms, regardless of the dimension. The compliance in completing the entire questionnaire was high at baseline (89 and 90% in experimental and standard regimen arms, respectively), then reduced during treatment and follow-up. Some missing items led to a lower compliance for dimension QL than for the others (for example, at baseline: 83% for QL vs. 89% for PF and 88% for PA and FA in the experimental regimen arm, and 86% for QL vs. 90% for PF, PA and FA in the standard regimen arm) (see Supplementary Figure 1).

#### Definition of the patterns for the PMM approach

We defined four patterns of dropout with well balanced effectives and a reasonable number of patients by pattern as well as clinically pertinent (see Fig. 1).

The first pattern grouped the patients who dropped out before visit V3 (last HRQoL measurement at V0, V1, or V2), that is, during or just after the period of radiochemotherapy and chemotherapy treatment. The patients who dropped out between V3 and V5 (last measurement at V3 or V4) formed the second pattern, and between V5 and V6 (last measurement at V5) the third pattern. The last pattern grouped the patients who dropped out between V6 and V7 (last measurement at V6) and the patients who did not drop out. For the QL dimension for example, the 252 evaluable patients were distributed as follows: 89/252 (*π*_1_ = 35%), 70/252 (*π*_2_ = 28%), 58/252 (*π*_3_ = 23%), and 35/252 (*π*_4_ = 14%) in the four respective patterns (for the other dimensions, see Fig. 1).

**Fig. 2:**
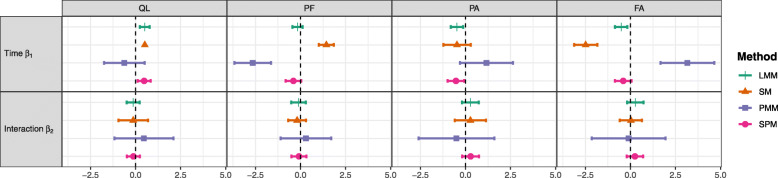
Estimated parameters and 95% confidence intervals. Legend: Time effect *β*_1_ (slope in the control arm) and interaction effect *β*_2_ (slope difference between the experimental and control arm) for the four dimensions of the EORTC QLQ-C30 (QL, PF, PA, and FA) according to the LMM, PMM, SM, SPM

The results of the longitudinal analysis of the QL, PF, PA, and FA scales of the EORTC QLQ-C30 using the four previously described approaches are summarized in Table 2 (estimates, 95% confidence intervals, and associated *p*-values of the Wald test) and graphically represented in Fig. 2 (estimated slope ${\hat{\beta}}_1$ and interaction ${\hat{\beta}}_2$ parameters).

**Table 2:** Results of the longitudinal analysis of the four dimensions of interest of the EORTC QLQ-C30

	LMM				SM				PMM				SPM			
		Estimate	CI 95%	*p*		Estimate	CI 95%	*p*		Estimate	CI 95%	*p*		Estimate	CI 95%	*p*
**QL** (*N* = 252)																
**Longitudinal outcome**	*β*_1_	0.513	[0.24; 0.78]	<0.001	*β*_1_	0.513	NA	NA	*β*_1_	−0.628	[−1.75; 0.50]	0.275	*β*_1_	0.477	[0.12; 0.84]	0.009
	*β*_2_	−0.130	[− 0.49; 0.23]	0.472	*β*_2_	− 0.130	[− 0.95; 0.69]	0.757	*β*_2_	0.464	[−1.17; 2.10]	0.578	*β*_2_	−0.122	[− 0.48; 0.24]	0.506
**Dropout**					*ψ*_0_	−0.808	[−1.31; − 0.31]	0.001	${\beta}_1^1$	−2.611	[−5.59; 0.37]	0.086	*γ*	−0.042	[− 0.29; 0.21]	0.744
					*ψ*_1_	−0.005	[− 0.01; 0.00]	0.221	${\beta}_2^1$	1.256	[−3.17; 5.69]	0.579	*α*	−0.003	[−0.02; 0.02]	0.756
					*ψ*_2_	0.005	[0.00; 0.01]	0.075	${\beta}_1^2$	−0.649	[−2.02; 0.72]	0.353	*ξ*_1_	0.110	[−3.46; 3.68]	<0.001
									${\beta}_2^2$	0.722	[−0.96; 2.41]	0.401	*ξ*_2_	0.105	[−3.50; 3.71]	<0.001
									${\beta}_1^3$	−0.009	[− 0.69; 0.67]	0.980	*ξ*_3_	0.181	[−3.45; 3.81]	0.006
									${\beta}_2^3$	−0.248	[−1.14; 0.64]	0.586	*ξ*_4_	0.122	[−3.52; 3.77]	0.001
									${\beta}_1^4$	0.477	[0.15; 0.81]	0.005	*ξ*_5_	0.06	[−3.65; 3.77]	<0.001
									${\beta}_2^4$	−0.123	[− 0.54; 0.29]	0.559	*ξ*_6_	0.065	[−3.76; 3.89]	<0.001
													*ξ*_7_	0.071	[−4.04; 4.18]	<0.001
**PF** (*N* = 254)																
**Longitudinal outcome**	*β*_1_	−0.164	[−0.45; 0.12]	0.266	*β*_1_	1.434	[1.02; 1.85]	<0.001	*β*_1_	−2.652	[−3.67; −1.64]	< 0.001	*β*_1_	−0.394	[− 0.83; 0.04]	0.078
	*β*_2_	−0.112	[− 0.51; 0.29]	0.586	*β*_2_	−0.189	[− 0.68; 0.31]	0.456	*β*_2_	0.300	[−1.10; 1.70]	0.676	*β*_2_	−0.084	[− 0.50; 0.33]	0.691
**Dropout**					*ψ*_0_	−3.231	[−5.16; −1.30]	0.001	${\beta}_1^1$	−6.526	[−9.16; − 3.89]	<0.001	*γ*	− 0.003	[−0.26; 0.25]	0.980
					*ψ*_1_	−0.089	[−0.11; − 0.06]	<0.001	${\beta}_2^1$	1.135	[−2.86; 5.13]	0.578	*α*	−0.015	[− 0.03; 0.00]	0.006
					*ψ*_2_	0.107	[0.07; 0.14]	<0.001	${\beta}_1^2$	−1.805	[−2.98; −0.63]	0.003	*ξ*_1_	0.272	[−2.88; 3.42]	0.006
									${\beta}_2^2$	0.362	[−1.15; 1.88]	0.640	*ξ*_2_	0.270	[−2.88; 3.42]	0.006
									${\beta}_1^3$	−0.474	[−1.06; 0.11]	0.115	*ξ*_3_	0.474	[−2.66; 3.61]	0.112
									${\beta}_2^3$	0.017	[−0.81; 0.84]	0.968	*ξ*_4_	0.309	[−2.82; 3.44]	0.012
									${\beta}_1^4$	0.152	[−0.16; 0.46]	0.340	*ξ*_5_	0.158	[−2.90; 3.22]	<0.001
									${\beta}_2^4$	−0.185	[− 0.61; 0.24]	0.391	*ξ*_6_	0.165	[−2.78; 3.12]	< 0.001
													*ξ*_7_	0.185	[−2.94; 3.31]	< 0.001
**PA** (N = 254)																
**Longitudinal outcome**	*β*_1_	−0.472	[− 0.82; − 0.13]	0.008	*β*_1_	−0.472	[−1.23; 0.29]	0.224	*β*_1_	1.166	[−0.30; 2.63]	0.118	*β*_1_	−0.527	[− 0.99; − 0.07]	0.024
	*β*_2_	0.276	[−0.19; 0.74]	0.242	*β*_2_	0.276	[−0.59; 1.14]	0.531	*β*_2_	−0.501	[−2.60; 1.60]	0.640	*β*_2_	0.285	[−0.18; 0.75]	0.231
**Dropout**					*ψ*_0_	0	[− 0.20; 0.20]	1	${\beta}_1^1$	4.582	[0.54; 8.62]	0.027	*γ*	−0.012	[−0.27; 0.24]	0.927
					*ψ*_1_	0	[−0.01; 0.01]	1	${\beta}_2^1$	−2.505	[−8.47; 3.46]	0.411	*α*	−0.003	[−0.02; 0.01]	0.720
					*ψ*_2_	0	[−0.01; 0.01]	1	${\beta}_1^2$	0.281	[−1.52; 2.08]	0.759	*ξ*_1_	0.086	[−2.49; 2.66]	< 0.001
									${\beta}_2^2$	0.048	[−2.15; 2.25]	0.966	*ξ*_2_	0.086	[−2.50; 2.67]	<0.001
									${\beta}_1^3$	0.264	[−0.60; 1.13]	0.551	*ξ*_3_	0.152	[−2.41; 2.72]	<0.001
									${\beta}_2^3$	0.239	[−0.93; 1.41]	0.689	*ξ*_4_	0.098	[−2.44; 2.63]	<0.001
									${\beta}_1^4$	−0.511	[−0.93; − 0.09]	0.017	*ξ*_5_	0.053	[−2.42; 2.52]	< 0.001
									${\beta}_2^4$	0.267	[−0.27; 0.80]	0.328	*ξ*_6_	0.055	[−2.31; 2.43]	<0.001
													*ξ*_7_	0.058	[−2.41; 2.52]	< 0.001
**FA** (N = 254)																
**Longitudinal outcome**	*β*_1_	−0.514	[−0.86; −0.17]	0.003	*β*_1_	−2.484	[−3.12; −1.84]	<0.001	*β*_1_	3.157	[1.67; 4.65]	<0.001	*β*_1_	−0.399	[−0.86; 0.06]	0.089
	*β*_2_	0.275	[−0.18; 0.73]	0.239	*β*_2_	0.027	[−0.59; 0.64]	0.932	*β*_2_	−0.098	[−2.15; 1.95]	0.926	*β*_2_	0.249	[−0.21; 0.71]	0.288
**Dropout**					*ψ*_0_	−0.907	[−1.27; − 0.54]	<0.001	${\beta}_1^1$	8.574	[4.67; 12.48]	<0.001	*γ*	−0.019	[−0.27; 0.23]	0.883
					*ψ*_1_	0.047	[0.03; 0.06]	<0.001	${\beta}_2^1$	−0.436	[−6.28; 5.41]	0.884	*α*	0.006	[−0.01; 0.02]	0.299
					*ψ*_2_	−0.097	[− 0.12; − 0.07]	<0.001	${\beta}_1^2$	3.118	[1.39; 4.85]	<0.001	*ξ*_1_	0.068	[−2.52; 2.66]	<0.001
									${\beta}_2^2$	−1.12	[−3.29; 1.05]	0.312	*ξ*_2_	0.064	[−2.55; 2.68]	<0.001
									${\beta}_1^3$	0.626	[−0.20; 1.45]	0.139	*ξ*_3_	0.117	[−2.48; 2.71]	<0.001
									${\beta}_2^3$	−0.087	[−1.21; 1.04]	0.880	*ξ*_4_	0.076	[−2.49; 2.64]	<0.001
									${\beta}_1^4$	−0.737	[−1.14; − 0.34]	<0.001	*ξ*_5_	0.041	[−2.48; 2.56]	<0.001
									${\beta}_2^4$	0.382	[−0.14; 0.90]	0.149	*ξ*_6_	0.045	[−2.39; 2.48]	<0.001
													*ξ*_7_	0.049	[−2.48; 2.58]	<0.001

Legend: *LMM* Linear Mixed Model, *SM* Selection Model, *PMM* Pattern-Mixture Model, *SPM* Shared-Parameter Model, *CI* Confidence Interval, *QL* Global Health Status, *PF* Physical functioning, *PA* Pain, *FA* Fatigue

No significant treatment-by-time interaction effect *β*_2_ was exhibited by the LMM. This was also the case for the SM, PMM, and SPM that had taken into account the dropout. Thus, none of the models suggested a significant effect of the treatment on the score evolution of the QL, PF, PA, and FA scales. The interaction parameters for the LMM (QL: ${\hat{\beta}}_2=-0.130$; PF: ${\hat{\beta}}_2=-0.112$; PA: ${\hat{\beta}}_2=0.276$; and FA: ${\hat{\beta}}_2=0.275$) were very close to those for the SM and SPM for all four dimensions. The interaction estimates from the PMM differed greatly from those of the other methods (QL: ${\hat{\beta}}_2=0.464$; PF: ${\hat{\beta}}_2=0.300$; PA: ${\hat{\beta}}_2=-0.501$; and FA: ${\hat{\beta}}_2=-0.098$) but also showed a greater uncertainty (larger confidence intervals).

The LMM showed a significant time effect for three of the four dimensions. More precisely, this model showed an increase in scale QL (${\hat{\beta}}_1=0.513,\kern0.5em p<0.001$) and a decrease in scales PA (${\hat{\beta}}_1=-0.472,\kern0.5em p=0.008$) and FA (${\hat{\beta}}_1=-0.514,\kern0.5em p=0.003$), reflecting a better level of HRQoL.

The SM confirmed or contradicted these results, depending on whether an association with the probability of dropout was detected or not. The SM and LMM estimated similar effects of time in the QL and in the PA scale where the dropout seemed to be ignorable (non-significant ${\hat{\psi}}_2$). However, there were unclear results with optimization difficulties: for scale QL, a numerical issue when inverting the Hessian matrix made it impossible to estimate the standard errors of ${\hat{\beta}}_1$, and therefore its confidence interval and associated *p*-value were not available; in view of the results for the PA scale, we could question whether or not the algorithm converged to a local minimum. When the SM detected an informative dropout (PF: ${\hat{\psi}}_2=0.107,\kern0.5em p<0.001$ and FA: ${\hat{\psi}}_2=-0.097,\kern0.5em p<0.001$), the estimated effect of time was larger than that estimated by the LMM, with a substantial increase in PF (SM: ${\hat{\beta}}_1=1.434,p<0.001$ vs. LMM: ${\hat{\beta}}_1=-0.164,p=0.266$) and decrease in FA (SM: ${\hat{\beta}}_1=-2.484,p<0.001$ vs. LMM: ${\hat{\beta}}_1=-0.514,p=0.003$). However, the values of ${\hat{\psi}}_2$ were counterintuitive, suggesting that the probability of dropout increased with an unobserved score value that corresponded to a higher level of HRQoL.

The marginal effect of time derived from the PMM estimates was ambiguous for all dimensions. For scales QL and PA, the direction of the time effect (i.e., the sign of ${\hat{\beta}}_1$) was reversed and no longer significant compared to the LMM. For the PF and FA scales, the HRQoL deterioration was aggravated compared to the LMM, with a significant increase in PF (PMM: ${\hat{\beta}}_1=-2.652,p<0.001$ vs. LMM: ${\hat{\beta}}_1=-0.164,p=0.266$) and FA (PMM: ${\hat{\beta}}_1=3.157,p<0.001$ vs. LMM: ${\hat{\beta}}_1=-0.514,p=0.003$), corresponding exactly with the same dimensions for which the SM had detected informative dropout.

**Fig. 3:**
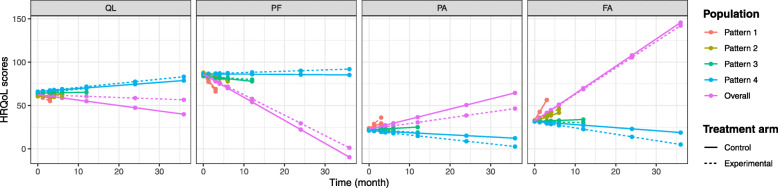
Predicted HRQoL score trajectories of the pattern-mixture model. Legend: Predictions over time by treatment arm regimen for the four dimensions of EORTC QLQ-C30 (QL, PF, PA, and FA). The linear trajectories are shown in pattern 1 (last measurement at visits V0 = 0, V1 = 1.25, or V2 = 3 months), pattern 2 (last measurement at visits V3 = 4 or V4 = 6 months), pattern 3 (last measurement at visits V5 = 12 months), pattern 4 (last measurement at visits V6 = 24 or V7 = 36 months, i.e., no dropout) and the overall patterns (marginal HRQoL scores). The solid line refers to the control fluorouracil-cisplatin regimen and the dashed line refers the experimental FOLFOX regimen

We observed that the estimated effect of time in the first pattern differed greatly from those in all other patterns (see also Fig. 3, which depicts the score trajectories by pattern).

The estimates in this pattern with a maximum of three repeated measures showed poor functional capacities (QL: ${\hat{\beta}}_1^1=-2.611,p=0.086$ and PF: ${\hat{\beta}}_1^1=-6.526,p<0.001$) and high levels of symptoms (PA: ${\hat{\beta}}_1^1=4.582,p=0.027$ and FA: ${\hat{\beta}}_1^1=8.574,p<0.001$). The estimates in this pattern were so important that they highly influenced the marginal estimates which could explain the difference in comparison with the other models.

As for the treatment-by-time interaction effect, we also observed that the 95% confidence intervals for the time effect were much larger than those seen in the other three models, reflecting more uncertainty.

For scales QL and PA, the estimated effect of time in the SPM was similar to that in the LMM. No association was detected between the risk of dropout and the current HRQoL score value, which confirmed the results of a non-informative dropout already identified by the SM. In contrast with the SM, the SPM also did not detect an association between the risk of dropout and the score in the FA scale, and the estimated time effect was similar to the LMM estimate. In fact, the SPM only detected a significant association between the risk of dropout and the score in the PF scale (also found by the SM) ($\hat{\alpha}=-0.015,p=0.006$). In particular, a decrease of 10 points in the PF score corresponded to a risk of dropout multiplied by 1.16 (95% confidence interval: [1.00, 1.35]). The estimation of the time effect was impacted (SPM: ${\hat{\beta}}_1=-0.394,p=0.078$ vs. LMM: ${\hat{\beta}}_1=-0.164,p=0.266$). Finally, the SPM allowed a more detailed analysis of the dropout process. The baseline hazard function was high at the beginning of the study and then decreased over time for the four scales (see the ${\hat{\xi}}_1,\dots, {\hat{\xi}}_7$ estimates). Besides this, the arm effect *γ* in the survival model was always non-significant, which suggests that there was no difference in the risk of dropout between the treatment arms.

**Fig. 4:**
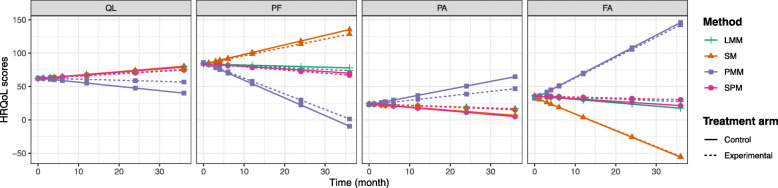
Predicted HRQoL score trajectories. Legend: Predictions for the four dimensions of the EORTC QLQ-C30 (QL, PF, PA, and FA) according to the LMM, PMM, SM, and SPM. The solid line refers to the control fluorouracil-cisplatin regimen and the dashed line refers the experimental FOLFOX regimen

Finally, Fig. 4 depicts how the differences between the models impacted the estimated HRQoL score trajectories.

The trajectories predicted by the PMM differed from the other models, showing poor functional capacities (QL and PF) and high levels of symptoms (PA and FA). The trajectories predicted by the SM contrasted with those of the PMM, particularly for scales PF and FA. Globally, the trajectories predicted by the SPM were consistent with those of the LMM.

## Discussion

Three approaches exist to model the joint distribution of a longitudinal outcome, such as a longitudinal score, and a dropout process: the SM, the PMM, and the SPM. In this article, we have compared them; firstly, from a methodological point of view, and secondly, when applied to data from the randomized clinical trial PRODIGE 5/ACCORD 17, which included 267 patients with advanced esophageal cancer. We have also compared the results of the three models with those obtained with the LMM.

All three approaches have different advantages and could be complementary. They also have different drawbacks and require assumptions that are untestable since they are based on unobserved data.

The PMM makes it possible to describe and study the HRQoL trajectories in each dropout pattern. In the application, the PMM revealed that the earlier the patients dropped out, the stronger their HRQoL deterioration. Besides this, by highlighting the different evolutions of HRQoL scores according to the dropout pattern, one can presume that the dropout process is informative. However, the PMM does not directly provide marginal estimates that would allow conclusions to be made for the whole population unless assumptions are made about the evolution of HRQoL trajectories after dropout. In our application we considered a simple PMM model with a linear HRQoL trajectory within each dropout pattern and a first pattern grouping patients with 1, 2 and 3 observations. It resulted in a direct and easy-to-implement formulation of the marginal estimates and implied that the HRQoL score evolution after dropout was extrapolated as an extension of the linear trajectories. This gave results that contradicted those obtained with the other models (the LMM, SM, and SPM) and with larger confidence intervals. Indeed, the first patterns including patients with few repeated measurements and a strong HRQoL deterioration highly influenced the marginal estimates. Note that in a more complex model, making identifying assumptions would be necessary [28]; a common strategy consists in using identifying restrictions [29]. Although unverifiable, the assumptions necessary to achieve identifiability in the PMMs and obtain marginal estimates have the advantage of being explicit.

The SM and SPM are interesting approaches because they can test the mechanism of missing data through interpretable parameters obtained from the logistic regression (SM) or the Cox model (SPM). In the application, when the dropout was detected as non-informative by the SM or the SPM the results for the trajectories of HRQoL were similar to those of the LMM and led to the same conclusions. Both models detected an informative dropout in the PF dimension but only the SM detected an informative dropout in the FA dimension. The SPM results were consistent with the LMM results and had a coherent interpretation. In contrast, the SM results revealed that the probability of dropout increased with an unobserved score value corresponding to a higher level of HRQoL. It is possible that these unexpected results are the consequence of the strong assumption of a normal distribution of the complete (observed and unobserved) HRQoL score values. Indeed, it has been shown that the SM is particularly sensitive to this unverifiable assumption [24, 30].

The SPM makes also modeling assumptions. In particular, it relies on the conditional independence between the longitudinal outcome and dropout process given the random effects. The random effects are also supposed to be normally distributed. Rizopoulos et al. showed that estimation of the parameters and standard errors could be sensible to misspecification of the random effects distribution, especially when some patients have very few measurements (early dropout) [31]. Note that in this application, we considered that the risk of dropout was associated with the HRQoL score through its current value. Other association structures could be considered, including the current slope or the random effects alone. The SPM alone is able to take into account dropout by modeling time-to-event data. Thus, unlike the PMM and the SM, the SPM treats the time-to-dropout as continuous. In our application, we used discrete dropout times corresponding to pre-specified assessment times, but the SPM would allow researchers to take into account dropouts corresponding to clinical events such as death, which can occur at any time between the HRQoL assessment times. By contrast, the use of the SPM was facilitated by the standard statistical software [27, 32–34]. Moreover, the existing programs allow for flexible models for the longitudinal outcome, more complex models for the time-to-dropout, and different association structures to capture the association between the longitudinal outcome and the time-to-dropout.

In this article, we have analyzed HRQoL data from the PRODIGE 5/ACCORD 17 clinical trial under three possible MNAR models accounting for informative dropout and the MAR corresponding model. MNAR methods, especially PMM, can also be used for sensitivity analysis to assess the robustness of the results [35].

This work has some limitations. The main objective was to compare MNAR models from a practical point of view but this does not allow to clearly decide between one model or the other. A simulation study would allow a comparison with statistical criteria by example in case of misspecification or by varying the proportion of missing data.

Longitudinal analysis of the HRQoL in the presence of missing data remains complex and unstandardized. Reviews and guidelines about reporting missing patient-reported outcome data in clinical trials have been published [36, 37]. It is recommended that the amount of missing data in each arm is reported and that the statistical methods used to handle missing data are explicitly specified. Nevertheless, there is no consensus for analyzing such data. Indeed, there is a lack of standardization and a gap between the development of statistical methods and their use in clinical trials [38, 39].

## Conclusions

This article aims to facilitate the understanding and use of such methods allowing analysis of longitudinal HRQoL data that include missing data due to dropout. Nevertheless, including in clinical trial protocol a plan to collect the reasons for non-responses would help to better characterize the missingness. Then, if informative dropout is suspected, we recommend using models that account for dropout, such as the SPM. In studies where no information is available on the reasons for missingness, the SPM can be used to confirm or invalidate the results of LMMs.

## Supplementary information

**Additional file 1: Figure S1.** Compliance in completing the EORTC QLQ-C30. Compliance in completing the entire questionnaire and for the four dimensions QL, PF, PA, and FA (ratio of the number of available questionnaires or scores to the number of expected questionnaires) at each HRQoL assessment visit (V) by treatment arm during radiochemotherapy (RT), chemotherapy (CT), and follow-up.

## Data Availability

The dataset analyzed during the current study is not publicly available due to confidentiality requirements. Data are however available from the main coordinator of the clinical trial, Pr Thierry Conroy, upon reasonable request, and with permissions of the study sponsor UNICANCER R&D.

## Abbreviations

EORTC: European Organisation for Research and Treatment of Cancer
FA: Fatigue
HRQoL: Health-related quality of life
LMM: Linear mixed model
MAR: Missing at random
MCAR: Missing completely at random
MNAR: Missing not at random
PA: Pain
PF: Physical functioning
PMM: Pattern-mixture model
QL: Global health status
QLQ-C30: Quality of Life Questionnaire Core 30
REML: Restricted maximum likelihood
SM: Selection model
SPM: Shared-parameter model
