# Supplementary material for: Handling informative dropout in longitudinal analysis of health-related quality of life: application of three approaches to data from the esophageal cancer clinical trial PRODIGE 5/ACCORD 17
Source: BMC Med Res Methodol. 2020 Sep 3;20:223. doi: 10.1186/s12874-020-01104-w (PMC7469318; doi:10.1186/s12874-020-01104-w)
Supplement: Supplementary file 1 — Additional file 1: Figure S1. Compliance in completing the EORTC QLQ-C30. Compliance in completing the entire questionnaire and for the four dimensions QL, PF, PA, and FA (ratio of the number of available questionnaires or scores to the number of expected questionnaires) at each HRQoL assessment visit (V) by treatment arm during radiochemotherapy (RT), chemotherapy (CT), and follow-up. [file 12874_2020_1104_MOESM1_ESM.pdf]

RT → CT → FOLLOW-UP

RANDOMIZED PATIENTS (N = 267)

QLQ-C30

	V0 Baseline	V1 Month 1.25	V2 Month 3	V3 Month 4	V4 Month 6	V5 Month 12	V6 Month 24	V7 Month 36
**FOLFOX REGIMEN (N = 134)**								
**QLQ-C30**	119/134 (89%)	92/133 (69%)	72/129 (56%)	48/124 (39%)	50/119 (42%)	48/101 (48%)	17/79 (22%)	5/67 (7%)
**QL**	111/134 (83%)	88/133 (66%)	67/129 (52%)	45/124 (36%)	45/119 (38%)	41/101 (41%)	15/79 (19%)	5/67 (7%)
**PF**	119/134 (89%)	92/133 (69%)	72/129 (56%)	48/124 (39%)	49 /119 (41%)	48/101 (48%)	17/79 (22%)	5/67 (7%)
**PA**	118/134 (88%)	92/133 (69%)	72/129 (56%)	48/124 (39%)	50/119 (42%)	48/101 (48%)	17/79 (22%)	5/67 (7%)
**FA**	118/134 (88%)	90 /133 (68%)	72/129 (56%)	48/124 (39%)	48/119 (40%)	48/101 (48%)	17/79 (22%)	5/67 (7%)
**FLUOROURACIL-CISPLATIN REGIMEN (N = 133)**								
**QLQ-C30**	120/133 (90%)	89/131 (68%)	62/126 (49%)	48/122 (39%)	46/117 (39%)	44/99 (44%)	17/70 (24%)	7/68 (10%)
**QL**	115/133 (86%)	82/131 (63%)	59/126 (47%)	43/122 (35%)	43/117 (37%)	40/99 (40%)	17/70 (24%)	7/68 (10%)
**PF**	120/133 (90%)	89/131 (68%)	62/126 (49%)	48/122 (39%)	46/117 (39%)	44/99 (44%)	17/70 (24%)	7/68 (10%)
**PA**	120/133 (90%)	89/131 (68%)	62/126 (49%)	48/122 (39%)	46/117 (39%)	43/99 (43%)	17/70 (24%)	7/68 (10%)
**FA**	120/133 (90%)	89/131 (68%)	62/126 (49%)	48/122 (39%)	46/117 (39%)	44/99 (44%)	17/70 (24%)	7/68 (10%)
